# Thermodynamic and kinetic stability of the Josephin Domain closed arrangement: evidences from replica exchange molecular dynamics

**DOI:** 10.1186/s13062-016-0173-y

**Published:** 2017-01-19

**Authors:** Gianvito Grasso, Jack A. Tuszynski, Umberto Morbiducci, Ginevra Licandro, Andrea Danani, Marco A. Deriu

**Affiliations:** 1 Istituto Dalle Molle di studi sull’Intelligenza Artificiale (IDSIA), Scuola universitaria professionale della Svizzera italiana (SUPSI), Università della Svizzera italiana (USI), Centro Galleria 2, Manno, CH-6928 Switzerland; 2 Department of Physics, University of Alberta, Edmonton, AB Canada; 3 Department of Mechanical and Aerospace Engineering, Politecnico di Torino, Corso Duca degli Abruzzi 24, IT-10128 Torino, Italy

**Keywords:** Ataxin, Replica exchange molecular dynamics, Neurodegenerative, Josephin Domain, Protein plasticity, Kinetics, Thermodynamics

## Abstract

**Background:**

Molecular phenomena driving pathological aggregation in neurodegenerative diseases are not completely understood yet. Peculiar is the case of Spinocerebellar Ataxia 3 (SCA3) where the conformational properties of the AT-3 N-terminal region, also called Josephin Domain (JD), play a key role in the first step of aggregation, having the JD an amyloidogenic propensity itself. For this reason, unraveling the intimate relationship between JD structural features and aggregation tendency may lead to a step forward in understanding the pathology and rationally design a cure. In this connection, computational modeling has demonstrated to be helpful in exploring the protein molecular dynamics and mechanism of action.

**Results:**

Conformational dynamics of the JD is here finely investigated by replica exchange molecular dynamics simulations able to sample the microsecond time scale and to provide both a thermodynamic and kinetic description of the protein conformational changes. Accessible structural conformations of the JD have been identified in: open, intermediate and closed like arrangement. Data indicated the closed JD arrangement as the most likely protein arrangement. The protein transition from closed toward intermediate/open states was characterized by a rate constant higher than 700 ns. This result also explains the inability of classical molecular dynamics to explore transitions from closed to open JD configuration on a time scale of hundreds of nanoseconds.

**Conclusion:**

This work provides the first kinetic estimation of the JD transition pathway from open-like to closed-like arrangement and vice-versa, indicating the closed-like arrangement as the most likely configuration for a JD in water environment. More widely, the importance of our results is also underscored considering that the ability to provide a kinetic description of the protein conformational changes is a scientific challenge for both experimental and theoretical approaches to date.

**Reviewers:**

This article was reviewed by Oliviero Carugo, Bojan Zagrovic.

**Electronic supplementary material:**

The online version of this article (doi:10.1186/s13062-016-0173-y) contains supplementary material, which is available to authorized users.

## Background

Protein conformational transition can be described as a complex search for a global energy minimum on a free energy surface, which depends on a huge number of molecular interactions and environmental factors [1–3]. In addition to their functional native states, globular proteins generally adopt intermediate conformational states corresponding to local minima on the free energy surface. Some of the energetically favorable alternatives may enable the exposure of hydrophobic protein domains, thus increasing the risk of both aberrant aggregation and related pathological transformations [4]. This is the case of amyloidogenic proteins, where a direct correlation between thermodynamic stability and propensity to amyloid fibril formation has been convincingly demonstrated [4, 5]. As a consequence, the kinetic and thermodynamic estimation of the protein conformational changes represents a significant scientific challenge and an important contribution to the comprehension of the molecular basis of amyloidogenic aggregation.

Recently, the conformational stability of the Josephin Domain (JD) has gained considerable attention in the research community. The JD is the N-terminal region of the Ataxin-3 (AT3) protein which is responsible for the spinocerebellar Ataxia 3 (SCA3) [6], a polyglutamine (polyQ) disease also known as Machado Joseph Disease (MJD).

Although in polyQ diseases the expanded polyQ tract is considered the main cause for protein misfolding and aggregation [7–16], the JD structural features play a pivotal role in driving the aggregation propensities and toxicity of AT3 protein [17–24]. In this regard, experimental studies have demonstrated that the first step of AT3 fibrillogenesis is JD-mediated [22, 25–27].

Several models of the Josephin Domain of Ataxin 3, solved by NMR have become recently available [20, 28–30]; however, these structures strongly differ for the hairpin conformation (region α2-α3, residues Val31-Leu62). Indeed, whereas the 1YZB [28] and 2JRI [20] models show a hairpin region that protrudes out into solution, the “closed” 2AGA [29] and “half-closed” 2DOS [30] models are characterized by the hairpin packed against the protein globular structure [30]. These JD models have been the subject of the several computational and experimental studies [31–33]. A recent investigation on JD conformational changes using both Classical MD and Metadynamics has estimated the whole free energy profile of the JD, demonstrating the closed conformation as the most representative for JD in water environment [31].

Computational modeling has been confirmed as a powerful tool to acquire indications and suggest hypothesis to be further tested by experiments [34–46]. For example, in one of our recent work [47], it was highlighted the propensity of the JD region α4 (and in particular Leu84-Trp87) to undergo high conformational changes as a consequence of the JD-JD binding. In a greater detail, α4 conformational changes, with consequent exposure of α4, was detected. Interestingly, both the α4 helical loss and its solvent exposure were observed only after the binding event. On the basis of our *in silico* results, we hypothesized a double step process involved in JD dimerization. Moreover, we suggested that the peptides sequence Lue84-Trp87 may be relevant for aberrant aggregation in a second step of the JD-JD binding, whereas the first step is mainly mediated by other residues such as Arg101. In this connection a recent experimental work [27] highlighted a transient local unfolding of α4, and consequent exposure of backbone amides to the solvent, able to trigger the AT-3 aggregation.

In the present work, additional evidence of the thermodynamic stability of the JD closed-like conformation is provided as a result of an extensive computational investigation concerning the JD conformational changes by Replica Exchange Molecular Dynamics. Moreover, a kinetic estimation of the conformational transition between the JD open and closed arrangements is reported here. The importance of the presented results is also underscored by the computational effort needed to provide kinetic description of the protein conformational changes, a scientific challenge for both experimental and theoretical approaches to date.

## Methods

The 1YZB model [28, 33] was selected as starting structure for the present work. The 1YZB model was determined by NMR technique and deeply validated in literature [28, 33]. Moreover the 1YZB has been considered as starting structure in all previous computational investigations focused on the JD of At3 [20, 31, 32, 47, 48].

### Replica exchange molecular dynamics

The 1YZB model was solvated in a dodecahedron box where the minimum distance between the protein and the edge of the box was 1 nm, resulting in a molecular system of about 40,000 interacting particles. The net charge of the system was neutralized at 0.15 M NaCl concentration. Energy minimization (1000 steps of Steepest Descent algorithm) and 50 ps of MD simulation with a Berendsen barostat [49] and a v-rescale thermostat [50] were performed to equilibrate the system at 310 K and 1 atm with time constants of τ_T_ = 0.1 ps and of τ_P_ = 0.2 ps, respectively. Replica Exchange Molecular Dynamics (REMD) [51] was carried out to explore the conformational ensembles of the JD. In detail, 128 replicas were simulated for temperatures ranging from 300 to 602 K in the NVT ensemble, as in previous works [52–54]. Temperatures were distributed according to an exponential spacing law, as suggested by previous studies [55, 56], keeping the overlap of the potential energy distributions constant across the temperature space (Section S1.1 of Additional file 1). The exchange attempt time interval was set to 2 ps. Each replica was simulated for 50 ns, obtaining a cumulative simulation time of 6.4 μs. AMBER99-ILDN force-field [57–59] and water TIP3P model [60] were chosen to describe the system topology. Electrostatic interactions were calculated at every step with the Particle-Mesh Ewald method with a short-range electrostatic interaction cut off of 1.2 nm. A cut-off of 1.2 nm was also applied to Lennard-Jones interactions. The v-rescale thermostat [50] was used for each replica to keep temperature constant with a time constant of τ_T_ = 0.1. The LINCS algorithm [61] approach allowed to apply a 2 fs time step integration strategy. GROMACS 4.6 package was used for all MD simulations and data analysis [62]. AMBER99-ILDN force-field [57–59] and water TIP3P model [60] were chosen to describe the system topology. The Visual Molecular Dynamics (VMD) [63] package was used for the visual inspection of the simulated systems. Analysis of secondary structure (SS) dynamics was performed by applying the STRIDE software [64, 65]. GROMOS clustering approach [66] was applied to the Replica Exchange Molecular Dynamics trajectory at 310 K in order to get insight into the likelihood of JD conformational arrangements.

### Kinetic estimation

In order to obtain a reliable kinetic estimation of the JD conformational changes, the approach developed by van der Spoel and coworkers [67] was applied to the REMD trajectories to provide a kinetic description. In detail, let be *F(t)*to be a binary indicator able to properly identify the protein conformational transition (i.e., *F(t)* = 1 corresponds to folded state at time *t* and *F(t)* = 0 corresponds to unfolded state). Starting from the calculation of *F(t)* from each snapshot of the REMD trajectories, the reactive flux correlation for each trajectory *j* is given by:

1 $$ \frac{d{F}_j(t)}{dt}={k}_f{U}_j(t)-{k}_u{F}_jt $$

where *k*_*f*_ is the rate constant (*u* - > *f*), *k*_*u*_ is the rate constant (*f* - > *u*) and *U(t)* represents the unfolded state [1-*F*].

The rate constants are related to the activation energies *E*_*A*_ and prefactors *A*, according to:

2 $$ {\mathrm{k}}_{\mathrm{u}}={\mathrm{A}}_{\mathrm{u}}{\mathrm{e}}^{-{\upbeta \mathrm{E}}_{\mathrm{A}}^{\mathrm{u}}},{\mathrm{k}}_{\mathrm{f}}={\mathrm{A}}_{\mathrm{f}}{\mathrm{e}}^{-{\upbeta \mathrm{E}}_{\mathrm{A}}^{\mathrm{f}}} $$

where β = 1/KT.

Rewriting Eq. 1 with the explicit time dependence of the temperature yields:

3 $$ \frac{d{F}_j(t)}{dt}={A}_f{e}^{-{\beta}_j(t){E}_A^f}{U}_j(t)-{A}_u{e}^{-{\beta}_j(t){E}_A^u}{F}_j(t) $$

Introducing *φ* as the integral of Eq. 3, the average over all trajectories, and a fitting parameter *Χ* ^2^ gives

4 $$ \varphi (t) = \frac{1}{N}{\displaystyle {\sum}_{j=1}^N}{\displaystyle \underset{0}{\overset{t}{\int }}}\frac{d{F}_j\left(\tau \right)d\tau }{d\tau } $$

5 $$ {X}^2=\frac{1}{N}{\displaystyle {\sum}_{j=1}^N}\ {\left[\varphi (t)-F(t)\right]}^2 $$

Then, the kinetics parameters *E*_*A*_^*u*^, *E*_*A*_^*f*^, *A*_*f*_, *A*_*u*_ were obtained by numerically minimizing the functional *Χ* ^2^. A detailed description of the method and parameters is reported in a previous publication [67].

In the present work, the description of the JD conformational space was carried out by using two different indicators already known to be appropriate for describing the JD transition pathway, (1) the Radius of Gyration (RG) and (2) the distance between regions α3 (Asp57-Leu62) and α5 (Pro97-Arg101) [31, 32, 47, 48]. In this manner, an estimation of the forward and backward rate constants for JD conformational transition, together with the activation energies, was obtained at 310 K [67].

## Results

**Fig. 1:**
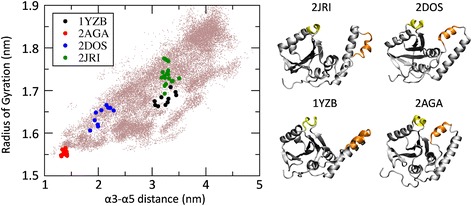
Each snapshot, taken from the entire REMD trajectory at 310 K, is reported in figure (*left*) in term Radius of Gyration and α3-α5 distance. The NMR snapshots derived from the JD models available in literature are colored in *red* (2AGA), *blue* (2DOS), *black* (1YZB) and *green* (2JRI). Each NMR model is also represented in figure (*right*). Secondary structures α3 (Asp57-Leu62) and α5 (Pro97-Arg101) are highlighted in *orange* and *yellow*, respectively

From the REMD simulations, (1) the temperature space was widely explored by each replica (meaning that each replica explored all temperature range) and (2) average acceptance ratio higher than 0.35 was obtained. Notably, it was found that JD secondary structure was highly conserved along the REMD trajectory at 310 K (Section 1.1 of Additional file 1), with the exception of α2 and (partially) α3, in agreement with the data reported earlier in the literature [31, 47]. The analysis of the scatter plot representing each REMD trajectory snapshot in term of Radius of Gyration and α3-α5 distance (Fig. 1), clearly demonstrates that several JD structural conformations are sampled along the REMD trajectory at 310 K.

In agreement with a very recent *in silico* study [31], 2AGA, 2DOS and 2JRI models lie, in terms of employed descriptors, in regions regularly sampled by REMD, differently from what observed in case of 1YZB model. It is worth mentioning that these observations are in close agreement with both classical MD and metadynamics simulations [31]. In this regard, it is also necessary to clarify that the findings of this study should not be intended as a quality check of 1YZB model, which has been already performed by applying appropriate methodologies [33]. Nonetheless the findings of this study might suggest that the half-open structure is stabilized under specific conditions, e.g., the presence of an interacting protein. This hypothesis needs to be carefully evaluated by a dedicated computational exploration, by modeling the presence of ubiquitin or of specific environmental conditions (ion concentration, protonation state, temperature, etc.).

**Fig. 2:**
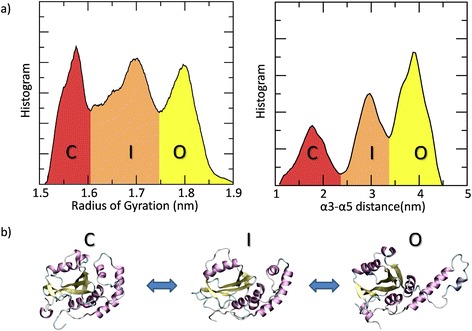
**a** Distribution of the JD radius of gyration (*left*) and α3-α5 distance (*right*) calculated for the REMD trajectories at 310 K. The closed (C), intermediate (I) and open (O) states are highlighted in *red*, *orange* and *yellow*, respectively. **b** Visual inspection of the JD conformational arrangements corresponding to closed (C), intermediate (I) and open (O) state, respectively

In the corresponding distribution plot (Fig. 2), it is possible to appreciate that the JD conformational space can be divided into three main groups: open (O) JD (RG around 1.81 nm and α3-α5 distance around 3.9 nm);closed (C) JD (RG value around1.56 nm and α3-α5 distance around 1.7 nm);intermediate (I) state (RG value around 1.70 nm and α3-α5 distance around 3.0 nm), containing both half-open and half-closed JD (Fig. 2a).

From the visual inspection of the JD arrangement corresponding to the mentioned conformational states (Fig. 2b), it can be observed that the centroid of the most populated cluster (obtained by using GROMOS [66] structure-based clustering approach) corresponds to the distribution peaks reported in Fig. 2a.

**Fig. 3:**
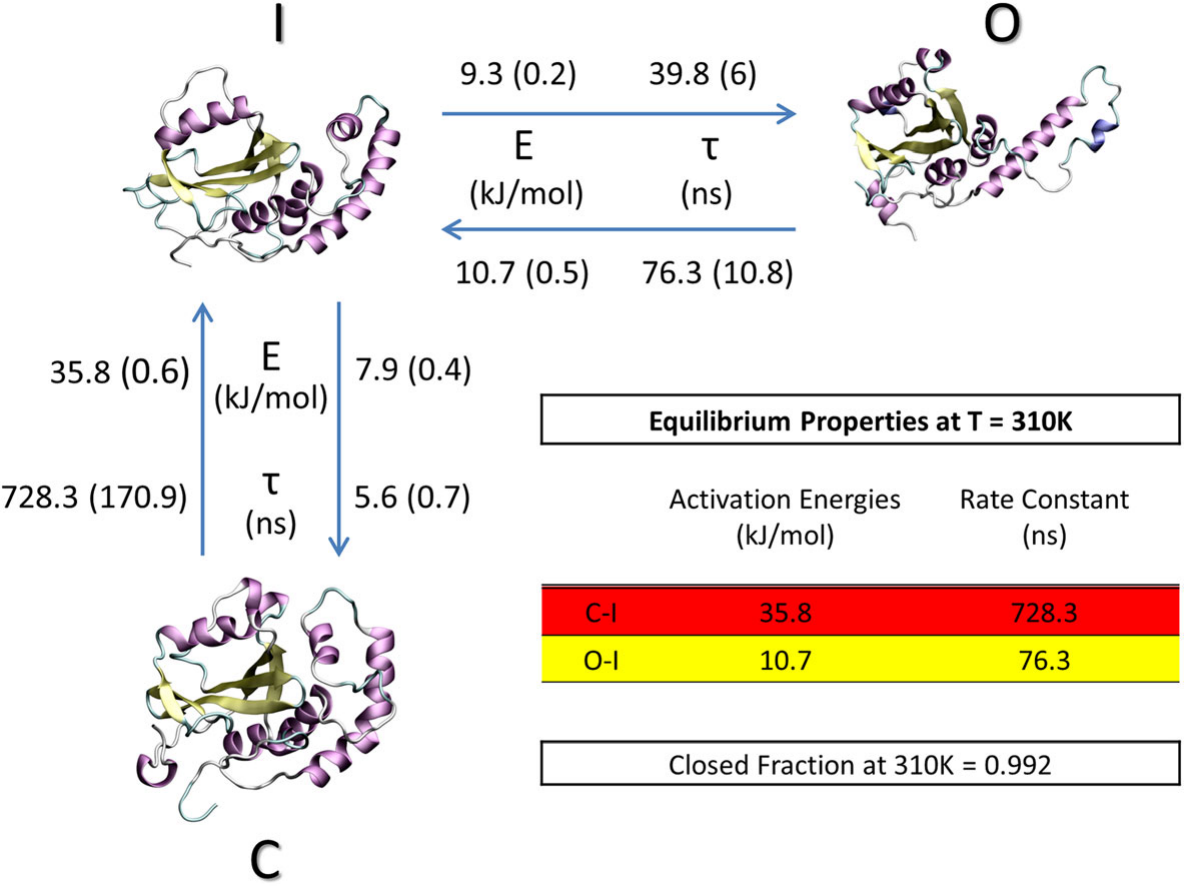
Visual inspection of the JD conformational arrangements corresponding to closed (C), intermediate (I) and open (O) state together with the estimated forward and backward rate constants for folding, together with activation energy values at 310 K. The kinetic and thermodynamic results shown in Fig. 3 are computed following the procedure reported in the Methods section of our paper, and implemented in the g_kinetics tool of GROMACS package. It is worth mentioning that the energy barrier reported in Fig. 3 represents free energy values of activation, containing also information about the entropic contribution. Errors estimate is also presented in *brackets*. Error analysis was performed by varying the cutoff distance used to define the protein folded state (open, intermediate or closed), as in previous works [67]. As pointed out Rhee and coworkers [73], the largest error is mainly related to the definition of what is folded [73]. In detail, a kinetic analysis was carried out by varying of 0.02 nm the Radius of Gyration threshold to discern between open/intermediate, and intermediate/closed

The kinetic and thermodynamic results shown in Fig. 3 are computed following the procedure reported in the Methods section of our paper, and implemented in the g_kinetics tool of GROMACS package. It is worth mentioning that the energy barrier reported in Fig. 3 represents free energy values of activation, containing also information about the entropic contribution. As a result, the closed JD arrangement represents the most energetically favorable configuration. In detail, transition between closed and intermediate states is characterized by a deep energy barrier (E_C-I_ = 35.8 ± 0.6 kJ/mol), in close agreement with the recently obtained computational results [31]. The corresponding rate constant is τ_C-I_ = 728.3 ± 170.9 ns, explaining the reason behind the well-established inability of the classical MD to explore the transition between closed and open JD on a time scale of hundreds of nanoseconds [31, 47]. On the other hand, the conformational change from the intermediate to the closed state is faster (τ_I-C_ = 5.6 ± 0.7 ns) and characterized by a lower energy barrier (E_I-C_ = 7.9 ± 0.4 kJ/mol). The energy barriers and rate constants between open (O) and intermediate (I) state are E_O-I_ = 10.7 ± 0.5 kJ/mol, τ_O-I_ = 76.3 ± 10.8 ns and E_I-O_ = 9.3 ± 0.2 kJ/mol, τ_I-O_ = 39.8 ± 6 ns, respectively (Fig. 3). Finally, the average JD closed fraction at 310 K, f_c_, estimated to a value of 0.992 (table of Fig. 3, and Section 1.2 of Additional file 1), provides further evidence for the closed arrangement as the most likely for a JD monomer alone in water environment.

## Discussion

A detailed thermodynamic and kinetic description of protein conformational transition could markedly enrich the arsenal of knowledge which is necessary to a deep comprehension of the molecular basis of amyloidogenic aggregation. Prompted by evidences indicating an intimate interconnection between the JD plasticity and AT-3 aggregation propensity [27], this work focused on the application of computational molecular modeling to investigate JD conformational transition from a kinetic and thermodynamic point of view. In a greater detail, the JD conformational accessible states were thoroughly sampled by REMD simulations. Investigating JD conformational plasticity might be a key to deeply understand JD amyloidogenic properties and successfully design novel strategies targeting SCA3.

In this connection, several issues have been addressed by our work as listed in the following.

### Kinetic estimation of Josephin Domain conformational transition

A kinetic description of the JD conformational changes is presented in this work, a novel aspect with respect to previous computational works focusing on JD conformational fluctuations [31, 32]. More specifically, kinetic data in terms of time constants, τ (Fig. 3), provide a further evidence that classical MD simulations may be unable to correctly sample the transition from JD closed to open conformations as also highlighted in previous studies [31, 47, 48]. Relevance of this kind of estimation is widely recognized, being protein conformational arrangements, in general, closely related to protein physiological function and a pathological behavior. This is also the case of the Josephin Domain. In this connection, kinetic analysis from our REMD simulations pointed out that, in absence of interacting proteins, the JD quickly (τ_I-O_ = 5.6 ± 0.7 ns) falls from an intermediate to a closed arrangement. This result has been confirmed also by a set of 100 independent unbiased MD (Additional file 1: S1.3). Instead the kinetics of the backward reaction is orders of magnitude slower (τ_O-I_ = 728.3 ± 170.9 ns).

### Evidence for a three state pathway and thermodynamic stability of the Josephin Domain

Our data depict the JD conformational transition as a 3-state pathway. Cluster analysis on REMD trajectories at 310 K has highlighted that JD conformations fall in three main classes: a closed arrangement (C), an intermediate state (I) and an open-like structure (O) as shown in Figs. 2 and 3. The above-mentioned conformational states can be considered as free energy local minima separated by energy barriers. The quantification of those barriers allowed us to gain information on thermodynamic stability of accessible JD arrangements. In a greater detail, data in Fig. 3 clearly show how thermal fluctuations might in principle provide the sufficient energy for driving an open-to-closed transition, moving through the intermediate state. Conversely, the reverse pathway is an unlikely event which necessitates an energy supply of an order of magnitude higher than thermal energy (310 K). It is opinion of the authors that our data do not exclude the existence of an open JD conformation which might be more likely under specific environmental conditions such as different pH value (which may cause protonation/deprotonation of specific protein residues). Within this framework, a recent work suggested [30] that structural variations of the JD arrangement may be due to the different experimental conditions [30], i.e., 298 K, pH 6.4 (2AGA [29]) or pH 6.5 (1YZB [28]) or 303 K, pH 7.0 (2DOS [30]). Moreover, the presence of JD functional partners, such as Ubiquitin protein, may change the JD conformational space and the related free energy landscape, leading to a higher stability of the JD open conformation. Our statement is supported by recent experimental works [21], showing that the interaction of JD with its physiological partner Ubiquitin strongly influences JD accessible conformations and aggregation propensity. In conclusion, information coming from our work, considering only the JD, together with further studies evaluating the influence of ubiquitin or other JDs on the JD conformational arrangements will greatly help in better understanding molecular reasons behind physiological function and pathological behavior. Nevertheless, the open, closed and intermediate models obtained in the present study may be considered as starting point for the above mentioned further computational investigations.

### Further evidences of the Josephin Domain closed arrangement stability

Here, REMD has been employed to explore accessible conformations of the JD of Ataxin 3. The obtained data strengthen the hypothesis of the closed-like arrangement as the most stable JD structure in water, as already suggested by previous investigations [31, 47]. It is interesting to notice that REMD simulations does not need any preliminary information regarding the molecular transition which were instead required to determine the collective variable in our previous work employing metadynamics driven by essential coordinates [31]. However, thermodynamics and kinetic analyses from REMD provided information on free energy barriers and time constants without depicting the overall free energy landscape shown in our previous work [31]. Taken together, the above mentioned computational evidences provide a complete and decisive picture of the conformational behavior characterizing the single JD of Ataxin 3 in water environment.

Further studies will involve the presence of interacting proteins, molecules, surfaces, or will consider specific point mutations. A change of the conformational space accessible to the JD [47, 48] and of the related free energy landscape is expected.

## Conclusions

The present study draws the attention on both thermodynamics and kinetic stability of the JD closed arrangement, which has been estimated to represent roughly 100% of the JD folded fraction (Fig. 3) at 310 K. The ability to provide a kinetic and thermodynamic description of the JD conformational changes is hoped to trigger further valuable advances in Ataxia research. For example, the approach here employed might be used to clarify the influence of small molecules, natural binders, and environmental factors, on the JD structural conformation. On the other hand, ambient conditions that have already shown to affect JD aggregation dynamics and kinetics (i.e., variation in pH or temperature, point mutations [26, 47], interaction with binders [21, 29, 68], interaction with surfaces [48]) might be simulated to verify their potential effect on JD structure. Given the already known intimate relationship between the JD structural plasticity and aggregation propensity [27], the identification of specific JD amyloidogenic conformations might open new routes for the design of novel rational drugs able to drive the JD thermodynamic and kinetic stability toward specific non-amyloidogenic conformations.

In conclusion, data presented in this study can be considered as a first important step showing a working methodology to be extended, in the future, to evaluate how physiological partners or designed compounds may influence the JD conformational arrangments from the thermodynamic and kinetic point of view. This information might be useful for a better understanding of the molecular reasons behind the JD aggregation propensity or for developing novel aggregation inhibitors.

## Reviewers’ comments

### Reviewer’s report 1: Oliviero Carugo, University of Vienna, Austria

Reviewer comments:

1. The manuscript submitted by Marco A. Deriu described a MD study of the Josephin domain of ataxin 3. With a computationally demanding Replica Exchange Molecular Dynamics, Deriu and colleagues were able to propose a mechanism of the open-close pathway and to estimate the thermodynamics and kinetics parameters of the path. Although interesting, this manuscript has a major problem. The open conformation of the 1YZB file of the Protein Data Bank was not observed amongst the computational models. Although the authors mention it in the manuscript, this is not enough. It is mandatory to examine in detail this discrepancy. It is possible that the NMR experimental structure is (partially) inaccurate. It is possible that the MD is (partially) inaccurate. It is possible that the physico-chemical conditions are different in the NMR experiment and in the simulation. Unless this point is completely clarified, I think that it is unnecessary to further review this manuscript.

**Fig. 4:**
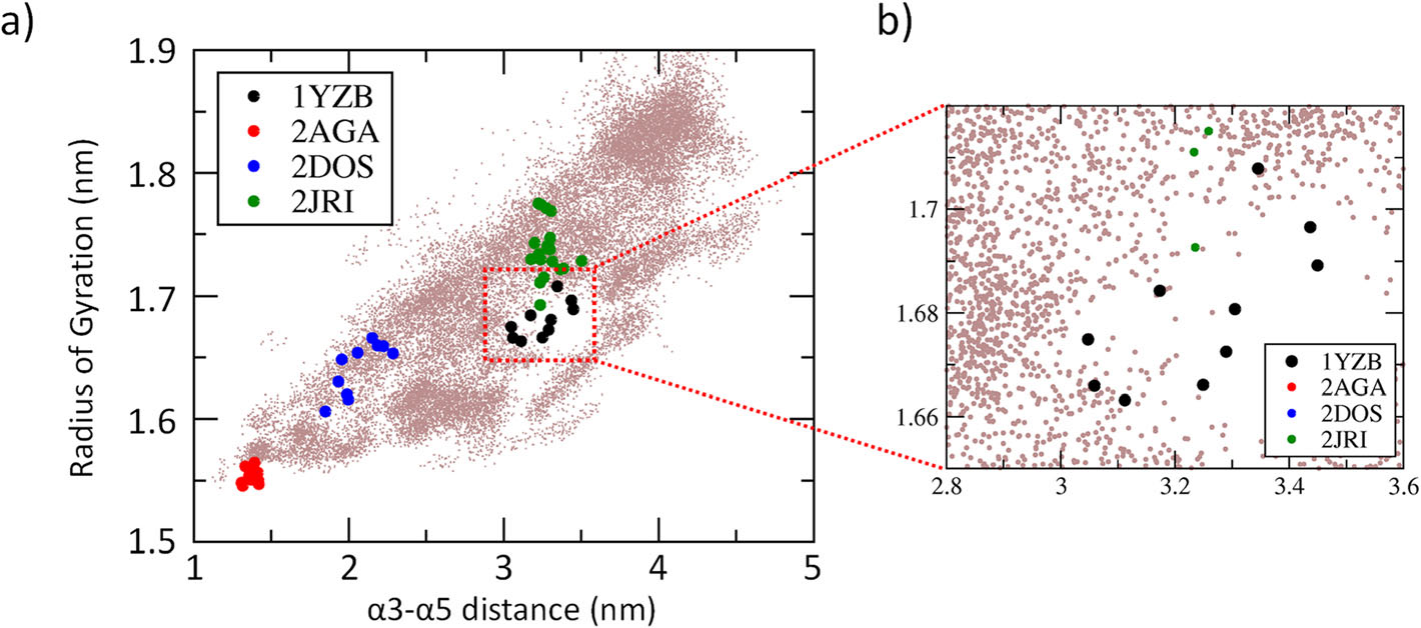
**a** Each snapshot, taken from the entire REMD trajectory at 310 K, is reported in figure in term Radius of Gyration and α3-α5 distance. The NMR snapshots derived from the JD models available in literature are colored in *red* (2AGA), *blue* (2DOS), *black* (1YZB) and *green* (2JRI). **b** A zoomed in view of the conformational sampling provided in the Figure a

Author’s response: *We thank the reviewer for highlighting this very important issue. The authors agree that it should be better emphasized in the manuscript. As a first point, the reviewer said that “* ***The open conformation of the 1YZB file of the Protein Data Bank was not observed amongst the computational models.*** *” However, the authors would like to specify that the 1YZB model, despite rarely sampled, is still observed throughout the REMD trajectory at 300 K (*Fig. 4 *). This is an extremely important point considering that from the theoretical point of view, in order to conclude that a specific protein conformational state has a lower free energy if compared with another one, the authors need to show not only that there is more sampling of one conformational state (*e.g.*, closed JD), but also that many transitions among the conformational states are sampled during the simulation. In this view, our simulations are conceived to sample many transitions among the JD accessible states, demonstrating that the closed-like arrangement is the most stable JD arrangement, from a thermodynamic and a kinetic point of view.*

As a second point, the reviewer said that “**It is mandatory to examine in detail this discrepancy. It is possible that the NMR experimental structure is (partially) inaccurate. It is possible that the MD is (partially) inaccurate. It is possible that the physico-chemical conditions are different in the NMR experiment and in the simulation.**”

For what concern the possible inaccuracy of the NMR experimental structures we would like to specify that our work is not oriented to demonstrate the “quality” of the 1YZB model, which was already evaluated in several high quality published experimental works [28, 33] in literature. On the other side, our MD simulations were performed following the state-of-the-art concerning atomistic Replica Exchange Molecular Dynamics protocols, which have widely demonstrated to be useful in describing the protein folding mechanism from the thermodynamic and kinetic point of view [39, 40, 55, 56, 69, 70].

However, the authors would like to better explain why 1YZB arrangement (open-like JD) is less sampled than the closed like JD. More than a limit of the work, this evidence represents in our opinion one of the most important results of the present work. It is opinion of the authors that our data do not exclude the existence of an open JD conformation which might be more likely under specific environmental conditions such as different pH value (which may cause protonation/deprotonation of specific protein residues). Within this framework, a recent work suggested [30] that structural variations of the JD arrangement may be due to the different experimental conditions [30], i.e., 298 K, pH 6.4 (2AGA [29]) or pH 6.5 (1YZB [28]) or 303 K, pH 7.0 (2DOS [30]).

More importantly, the presence of JD functional partners, such as Ubiquitin protein, may change the JD conformational space and the related free energy landscape, leading to a higher stability of the JD open conformation. Our assumption is supported by recent experimental work [20], showing that the interaction of JD with its physiological partner Ubiquitin strongly influences JD accessible conformations and aggregation propensity. We might expect a more sampled 1YZB in presence of ubiquitin. This investigation is a very demanding computational task and will be the subject of future research.

**Fig. 5:**
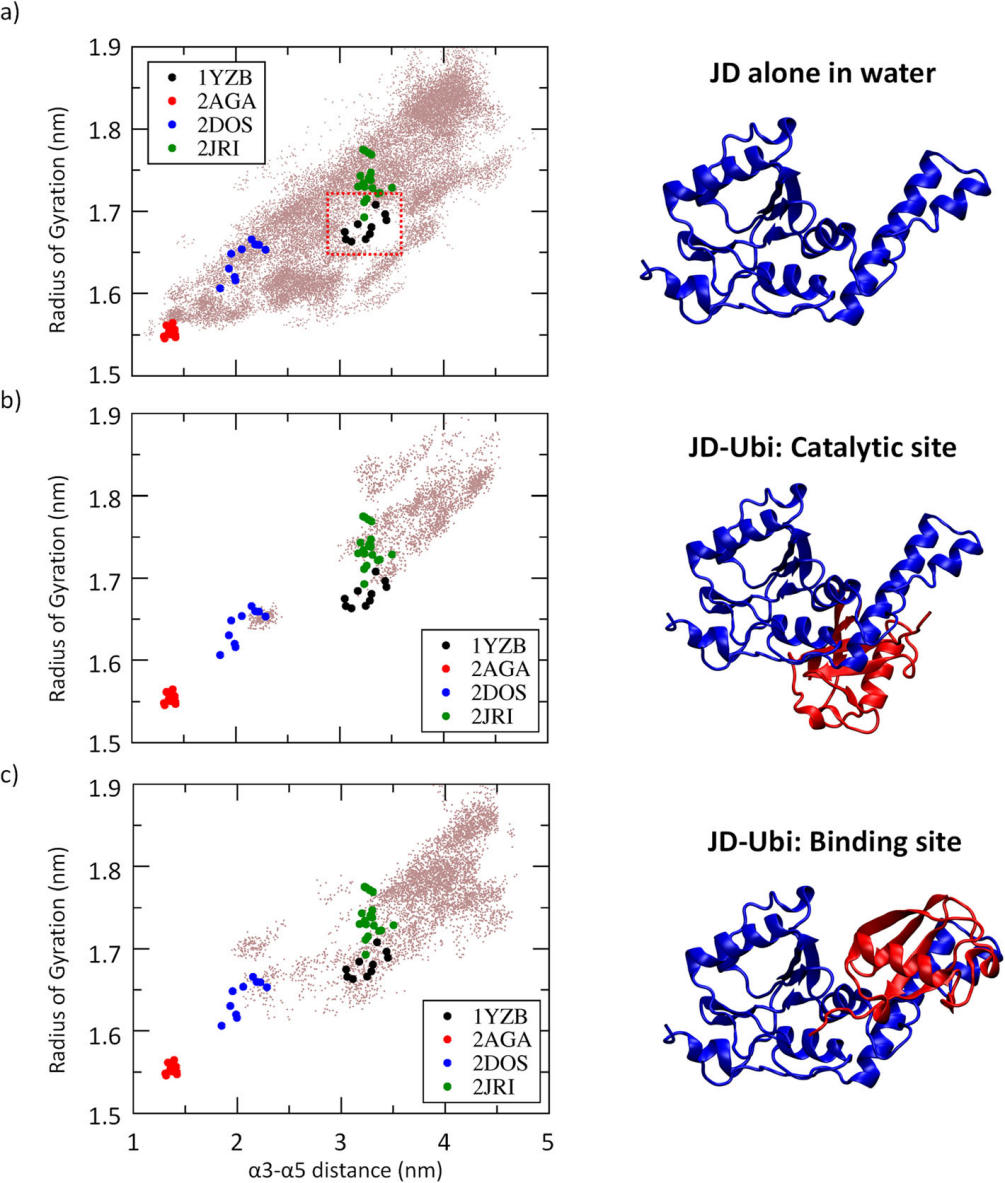
**a** Each snapshot, taken from the entire REMD trajectory at 310 K of the JD alone in water environment, is reported in figure in term Radius of Gyration and α3-α5 distance. The NMR snapshots derived from the JD models available in literature are colored in *red* (2AGA), *blue* (2DOS), *black* (1YZB) and *green* (2JRI). **b** Each snapshot, taken from the entire REMD trajectory at 310 K of the JD-Ubi complex (Ubiquitin located in the JD catalytic site), is reported in figure in term Radius of Gyration and α3-α5 distance. The NMR snapshots derived from the JD models available in literature are colored in *red* (2AGA), *blue* (2DOS), *black* (1YZB) and *green* (2JRI). **c** Each snapshot, taken from the entire REMD trajectory at 310 K of the JD-Ubi complex (Ubiquitin located in the JD binding site), is reported in figure in term Radius of Gyration and α3-α5 distance. The NMR snapshots derived from the JD models available in literature are colored in *red* (2AGA), *blue* (2DOS), *black* (1YZB) and *green* (2JRI)

However, in order to fully address the reviewer answer, we will show a preliminary REMD simulations starting from the atomic structure of the JD-Ubiquitin (JD-Ubi) complex obtained from RCSB Protein Data Bank (PDB entry 2JRI.pdb). The latter pdb file includes both the possible JD-Ubiquitin complexes: a) the JD-Ubi catalytic site and b) the JD-Ubi binding site. Each JD-Ubi model was solvated in a dodecahedron box where the minimum distance between the protein and the edge of the box was fixed as 1 nm, resulting in a molecular system of about 50,000 interacting particles. The same REMD procedure performed in the present work was applied to deeply sample the accessible JD conformational states in presence of the functional partner Ubiquitin. As a result, we are able to observe that the JD conformational states sampled along the REMD trajectory are strongly influenced by the interacting protein partner (Fig. 5). In detail, it is worth mentioning that the 1YZB model, less sampled in the REMD simulation of the JD protein alone in water environment, is more sampled during the JD-Ubi simulations. This result is particularly noticeable if we analyze the JD conformational states sampled with the Ubiquitin in the JD binding site (Fig. 5, bottom). Moreover, the completely closed protein conformation was never observed during the JD-Ubi simulations, demonstrating that the presence of interacting functional partner strongly stabilize the JD open conformation, as suggested in literature by experimental evidences [21].

The authors would consider the above presented novel results as part of a further work more oriented in investigating the relationship between JD conformation and JD interaction with protein “partners”. However, we are more than willing to insert this part into the manuscript if it is so desired by the reviewer.

Several sentences have been added in the revised version of the manuscript (Discussion section), as requested by the reviewer:

“It is opinion of the authors that our data do not exclude the existence of an open JD conformation which might be more likely under specific environmental conditions such as different pH value (which may cause protonation/deprotonation of specific protein residues). Within this framework, a recent work suggested [30] that structural variations of the JD arrangement may be due to the different experimental conditions [30], i.e., 298 K, pH 6.4 (2AGA [29]) or pH 6.5 (1YZB [28]) or 303 K, pH 7.0 (2DOS [30]). Moreover, the presence of JD functional partners, such as Ubiquitin protein, may change the JD conformational space and the related free energy landscape, leading to a higher stability of the JD open conformation. Our statement is supported by recent experimental works [21], showing that the interaction of JD with its physiological partner Ubiquitin strongly influences JD accessible conformations and aggregation propensity. In conclusion, information coming from our work, considering only the JD, together with further studies evaluating the influence of ubiquitin or other JDs on the JD conformational arrangements will greatly help in better understanding molecular reasons behind physiological function and pathological behavior.”

### Reviewer’s report 2: Bojan Zagrovic, Mediterranean Institute for Life Sciences, Croatia

Reviewer comments:

1. The authors use replica exchange molecular dynamics simulations to study to open-to-close conformational transition of the Josephin Domain (JD) of Ataxin 3. While technically solid and biologically relevant, the paper still suffers from multiple deficiencies, which should be addressed prior to publication. A major criticism concerns the presentation of the simulated trajectories and the associated rates as relating to “folding” of the JD. Namely, what the authors examine is just the very few last steps in the complete folding mechanism of the JD, the transition from the open to closed arrangement, and not nearly anything related to the folding process of the molecule starting from the unfolded state. The text should be accurately rephrased, starting with the title, to better reflect this fact.

Author’s response: *We thank the reviewer for highlighting this point. The authors agree that the protein conformational transition described in the present work from open to closed JD and viceversa, is not “* ***related to the folding process of the molecule starting from the unfolded state*** *”. For this reason, the authors propose to modify the title as follows:*

“Thermodynamic and Kinetic Stability of the Josephin Domain Closed Arrangement: Evidences from Replica Exchange Molecular Dynamics”

Following the suggestions of the Reviewer, we have also accurately rephrased the misleading sentences in the revised version of the manuscript, when appropriate.

Reviewer comments:

2. The main descriptors of conformational transition used in the analysis are radius of gyration and distance between regions alpha3 and alpha5. As one of the main challenges in the accurate determination of rates from folding simulations is the proper choice of definition of the folded and other states, the authors should analyze their trajectories from the perspective of other relevant order parameters, such as RMSD or number of native contacts.

**Fig. 6:**
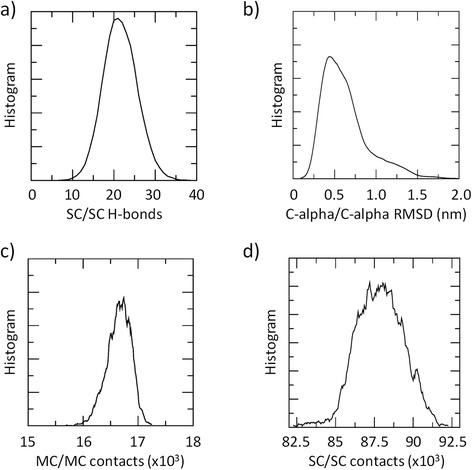
**a** Number of Side-Chain (SC)/Side-Chain (SC) H-bonds distribution calculated over the entire REMD trajectory at 310 K. **b** Distribution plot of the Root Mean Square Deviation (RMSD), computed over the entire REMD trajectory at 310 K. **c** Number of Main-Chain (MC)/Main-Chain (MC) H-bonds contacts calculated over the entire REMD trajectory at 310 K. **d** Number of Side-Chain (SC)/Side-Chain (SC) contacts distribution calculated over the entire REMD trajectory at 310 K

Author’s response: *As stated by the reviewer, finding proper collective variables might be nontrivial, and remains one of the most challenging issues in the field of enhanced sampling techniques. Among others, collective variables such as Root Mean Square Deviation* [71]*, number of native contacts* [71]*, and AlphaBeta similarities* [72] *have widely demonstrated to be useful in estimating the free energy landscape of protein folding. However, as stated by the reviewer (Please see also point 1), “* ***what the authors examine is just the very few last steps in the complete folding mechanism of the JD, the transition from the open to closed arrangement, and not nearly anything related to the folding process of the molecule starting from the unfolded state*** *”. In this specific case, the protein conformational transition from open to closed JD is a molecular event not directly related to the previously mentioned variables. This is demonstrated analyzing the distribution plots of the protein Side-Chain/Side-Chain H-bonds (*Fig. 6a *), the C-alpha/C-alpha Root Mean Square Deviation (*Fig. 6b *), the Main-Chain/Main-Chain contacts (*Fig. 6c *), and the Side-Chain/Side-Chain contacts (*Fig. 6d *) computed over the REMD trajectory at 310 K. In all cases, the distribution plots are characterized by a single dominant peak, demonstrating that the selected Collective Variable is not able to distinguish the different JD conformational arrangements in water environment. For this reason, the authors have used two collective variables (*i.e.*, Radius of Gyration and alpha3-alpha5 distance) given their ability to sample the JD conformational change from open to closed JD and viceversa, as demonstrated in* Fig. 2 *of the manuscript.*

Reviewer comments:

3. Also, the authors should provide a detailed sensitivity analysis concerning the dependence of the obtained rates on the exact definition of the relevant macrostates.

Author’s response: *The authors have analyzed the “* ***dependence of the obtained rates on the exact definition of the relevant macrostates”*** *following the procedure reported in the reference literature* [67, 73]*. In detail, the error value corresponding to each thermodynamic and kinetic quantity estimated in the manuscript is calculated by varying the cutoff for identifying the protein conformational states (open, intermediate or closed, respectively). The previously mentioned approach has already demonstrated to be appropriate in literature* [67]*, considering that the largest error is mainly related to the definition of the cutoff value* [73]*. In detail, we performed our kinetic analysis by varying of 0.02 nm the Radius of Gyration threshold to discern between open/intermediate, and intermediate/closed.* Figure 3 *of the manuscript has been modified accordingly.*

Reviewer comments:

4. The Methods sections should be expanded such as to include relevant technical details (type of thermostat and barostat, settings for the two, ion concentration, details of PME calculations etc.)

Author’s response: *The authors have added several sentences in the “Materials and Methods” section to include relevant technical details as requested by the reviewer:*

“The 1YZB model was solvated in a dodecahedron box where the minimum distance between the protein and the edge of the box was 1 nm, resulting in a molecular system of about 40,000 interacting particles. The net charge of the system was neutralized at 0.15 M NaCl concentration. Energy minimization (1000 steps of Steepest Descent algorithm) and 50 ps of MD simulation with a Berendsen barostat [49] and a v-rescale thermostat [50] were performed to equilibrate the system at 310 K and 1 atm with time constants of τ_T_ = 0.1 ps and of τ_P_ = 0.2 ps, respectively.”

[…]

“Each replica was simulated for 50 ns, obtaining a cumulative simulation time of 6.4 μs. AMBER99-ILDN force-field [57–59] and water TIP3P model [60] were chosen to describe the system topology. Electrostatic interactions were calculated at every step with the Particle-Mesh Ewald method with a short-range electrostatic interaction cut off of 1.2 nm. A cut-off of 1.2 nm was also applied to Lennard-Jones interactions. The v-rescale thermostat [50] was used for each replica to keep temperature constant with a time constant of τ_T_ = 0.1. The LINCS algorithm [61] approach allowed to apply a 2 fs time step integration strategy. GROMACS 4.6 package was used for all MD simulations and data analysis [62].”

Reviewer comments:

5. It is not clear how the energy barrier (p.7, l31) is evaluated. Is this a potential energy barrier or a free energy barrier?

Author’s response: *We thank the reviewer for highlighting this point. The energy barriers reported in (p.7, L31) are free energy values of activation that contain also information about the entropic contribution. The previously mentioned values are computed following the procedure reported in the “Thermodynamic and kinetic estimation” of the Materials and Methods section of our paper, and implemented in the g_kinetics tool of GROMACS package. In order to better clarify this point, the authors have added few sentences in the results section as well as in the Caption of* Fig. 3 *:*

Results section:

“The kinetic and thermodynamic results shown in Fig. 3 are computed following the procedure reported in the Materials and Methods section of our paper, and implemented in the g_kinetics tool of GROMACS package. It is worth mentioning that the energy barrier reported in Fig. 3 represents free energy values of activation, containing also information about the entropic contribution. As a result, the closed JD arrangement represents the most energetically favorable configuration. In detail, transition between closed and intermediate states is characterized by a deep energy barrier (E_C-I_ = 35.8 ± 0.6 kJ/mol), in close agreement with the recently obtained computational results [31].”

Caption Fig. 3:

“Visual inspection of the JD conformational arrangements corresponding to closed (C), intermediate (I) and open (O) state together with the estimated forward and backward rate constants for folding, together with activation energy values at 310 K. The kinetic and thermodynamic results shown in Fig. 3 are computed following the procedure reported in the Materials and Methods section of our paper, and implemented in the g_kinetics tool of GROMACS package. It is worth mentioning that the energy barrier reported in Fig. 3 represents free energy values of activation, containing also information about the entropic contribution. Errors estimate is also presented in brackets. Error analysis was performed by varying the cutoff distance used to define the protein folded state (open, intermediate or closed), as in previous works [67]. As pointed out Rhee and coworkers [73], the largest error is mainly related to the definition of what is folded [73]. In detail, a kinetic analysis was carried out by varying of 0.02 nm the Radius of Gyration threshold to discern between open/intermediate, and intermediate/closed.”

Reviewer comments:

6. The melting curve shown in Additional file 1: Figure S3 suggests that the protein remains well-folded (i.e. in a closed conformation) well over 400 K, and that its melting temperature is likely well over 500 or 600 K, which is clearly a physical impossibility. The authors should comment on this excessively high stability and link it with the potential methodological deficiencies.

**Fig. 7:**
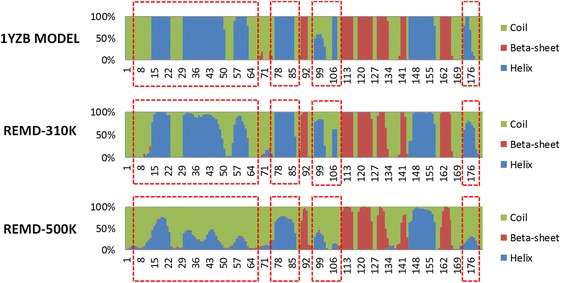
Secondary structure percentage, calculated in the following cases: i) over the available NMR configurations contained in 1YZB model, ii) over the REMD trajectory at 310 K, iii) over the REMD trajectory at 500 K. Secondary structures are indicated by different colors in the figure: *green* (coil), *blue* (helix), *red* (β-sheet)

Author’s response: *As previously mentioned (Please see point 1 and point 2 of Reviewer 2), the protein conformational transition described in the present work from open to closed JD and* vice-versa*, is not related to the folding process of the molecule starting from the unfolded state. In this view, the melting temperature reported in Additional file 1: Figure S3 does not provide information about the stability of the folded protein domain in water environment. In other words, the “melting curve” reported in Additional file 1: Figure S3 highlight that, even at high temperature (well over 400 K), the most likely protein conformational state is a closed-like arrangement. However, as expected, the protein fails to form its proper secondary structure at high temperature. This evidence is demonstrated comparing the secondary structure percentage of the available NMR configurations contained in 1YZB with the trajectory snapshots taken from the REMD trajectory at 310 K and at 500 K (*Fig. 7 *). Despite the protein secondary structures are well conserved in the REMD trajectory at 310 K, the JD protein fails to form its proper secondary structure at high temperature, as expected.*

Reviewer comments:

7. The written text, while as a whole acceptable, should still undergo a round of proofreading for grammatical and stylistic errors. Altogether, the article should provide more mechanistic details about the folding mechanism of JD.

Author’s response: *The text has been checked and corrected to the best of our ability, as suggested by the reviewer. The authors believe that the previous corrections and added comments will provide to the manuscript a more mechanistic view of the JD opening/closing dynamics.*

## Additional file

Additional file 1: Supporting Information to Thermodynamic and Kinetic Stability of the Josephin Domain Closed Arrangement: Evidences from Replica Exchange Molecular Dynamics. Additional Information on Replica Exchange applied approach, kinetic estimation. (PDF 1183 kb)

## Abbreviations

At3: Ataxin 3
JD: Josephin Domain
MD: Molecular dynamics
REMD: Replica exchange molecular dynamics
RG: Radius of gyration
