# Supplementary material for: Thermodynamic and kinetic stability of the Josephin Domain closed arrangement: evidences from replica exchange molecular dynamics
Source: Biol Direct. 2017 Jan 19;12:2. doi: 10.1186/s13062-016-0173-y (PMC5244572; doi:10.1186/s13062-016-0173-y)
Supplement: Additional file 1: — Supporting Information to Thermodynamic and Kinetic Stability of the Josephin Domain Closed Arrangement: Evidences from Replica Exchange Molecular Dynamics. Additional Information on Replica Exchange applied approach, kinetic estimation. (PDF 1183 kb) [file 13062_2016_173_MOESM1_ESM.pdf]

Supporting Information to Thermodynamic and Kinetic Stability of the Josephin Domain Closed Arrangement: Evidences from Replica Exchange Molecular Dynamics

Supporting Information

Gianvito Grasso[1], Jack A. Tuszynsy[2], Umberto Morbiducci[3], Ginevra Licandro[1], Andrea Danani[1], and Marco A. Deriu[1*]*

* *Gianvito Grasso and Marco A. Deriu equally contributed to the work.*

[1] Istituto Dalle Molle di studi sull'Intelligenza Artificiale (IDSIA), Scuola universitaria professionale della Svizzera italiana (SUPSI), Università della Svizzera italiana (USI), Centro Galleria 2, Manno, CH-6928, Switzerland.

[2] Department of Physics, University of Alberta, Edmonton AB, Canada.

[3] Department of Mechanical and Aerospace Engineering, Politecnico di Torino, CorsoDucadegli Abruzzi 24, IT-10128, Torino, Italy.

corresponding author: Marco A. Deriu: marco.deriu@idsia.ch

Supporting Information to Thermodynamic and Kinetic Stability of the Josephin Domain Closed Arrangement: Evidences from Replica Exchange Molecular Dynamics

Section 1

S1.1 Replica Exchange Molecular Dynamics

The 1YZB model [1,2] of JD was selected as starting point for the present work. The 1YZB model was solvated in a dodecahedron box where the minimum distance between the protein and the edge of the box was 1 nm, resulting in a molecular system of about 40000 interacting particles. 128 replicas were simulated for temperatures ranging from 300 K to 602 K in NVT ensemble, as done in several works in literature [3–5]. Temperatures are distributed following the exponential spacing law suggested by a number of paper [6,7], keeping the overlap of the potential energy distributions constant across the temperature space (Figure S2). The resulting exchange probability was 0.35. The exchange attempt time interval was set to 2 ps. Each replica was simulated for 50 ns, obtaining a cumulative simulation time of 6.4 µs. It is worth noticing that JD secondary structure is highly conserved along the REMD trajectory at 310K (Figure S2), with the exception of α2 and (partially) α3, in agreement with data coming from literature [8,9]

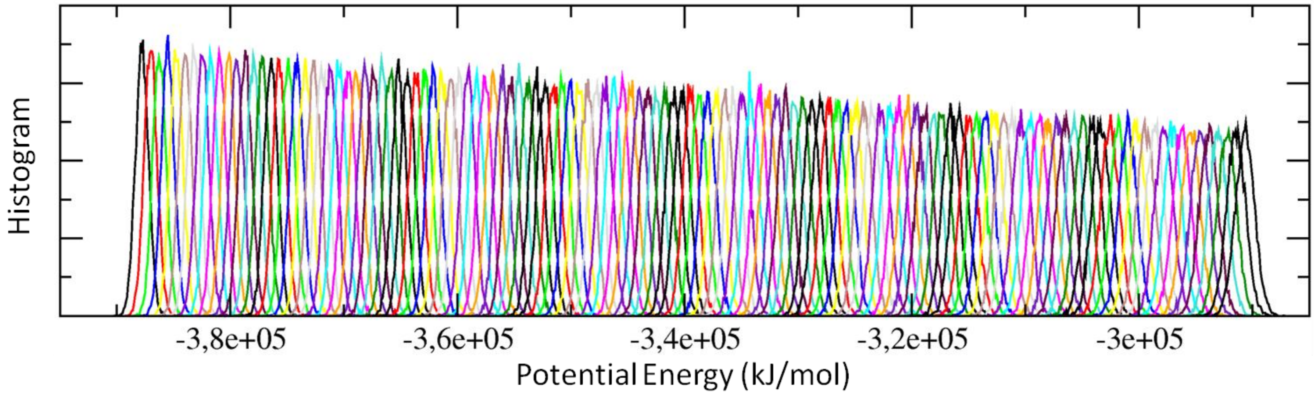

Figure S1. Potential energy distribution among REMD simulations. Temperatures were distributed across the replicas in a geometric progression, i.e. with the same ratio used to scale each temperature from the one below it, keeping the overlap of the potential energy distributions constant across the temperature space.

Supporting Information to Thermodynamic and Kinetic Stability of the Josephin Domain Closed Arrangement: Evidences from Replica Exchange Molecular Dynamics

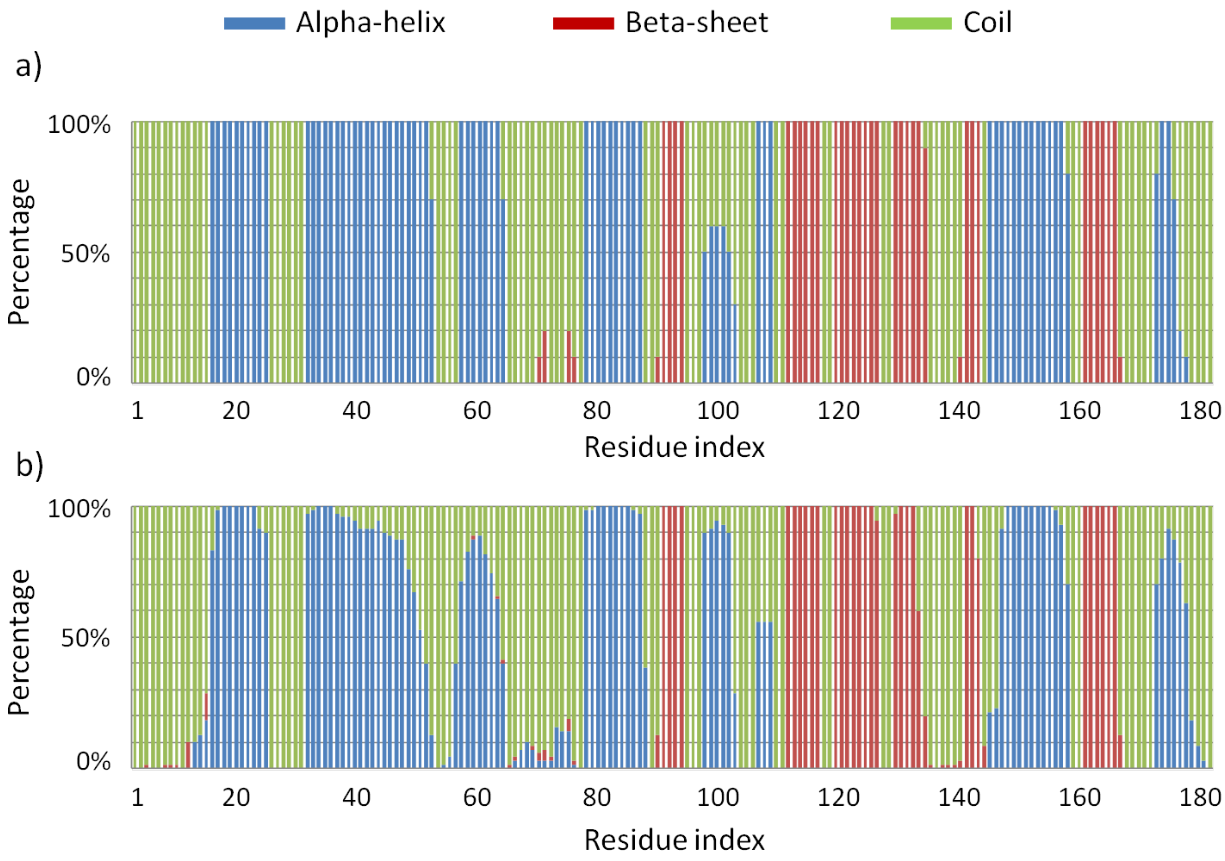

Figure S2. Secondary structure probability calculated in case of a) Josephin Domain 1YZB model [1,2] compared with b) REMD trajectory snapshots at 310 K. JD secondary structure is highly conserved along the REMD trajectory at 310K, with the exception of α2 and partially α3.

Supporting Information to Thermodynamic and Kinetic Stability of the Josephin Domain Closed Arrangement: Evidences from Replica Exchange Molecular Dynamics

S1.2 Thermodynamic and Kinetic Estimation

In order to obtain a reliable estimate of the JD folding rates, the kinetic description developed by van der Spoel et al [10] was applied to the REMD trajectories, as described in the main text of this paper. The fraction of closed JD sampled along the REMD trajectories is reported in Figure S3a as function of simulation time. The red curve of Figure S3a is optimized according to Equation 5 reported in the main text of the present paper. This function depends on the mix of simulations and temperatures used in the analysis, and the results show that the red curve represents a good fit of the computational data (Figure S3a). From these data, together with the estimation of the forward and backward rate constants for folding, a theoretical melting curve was obtained (Figure S3b).

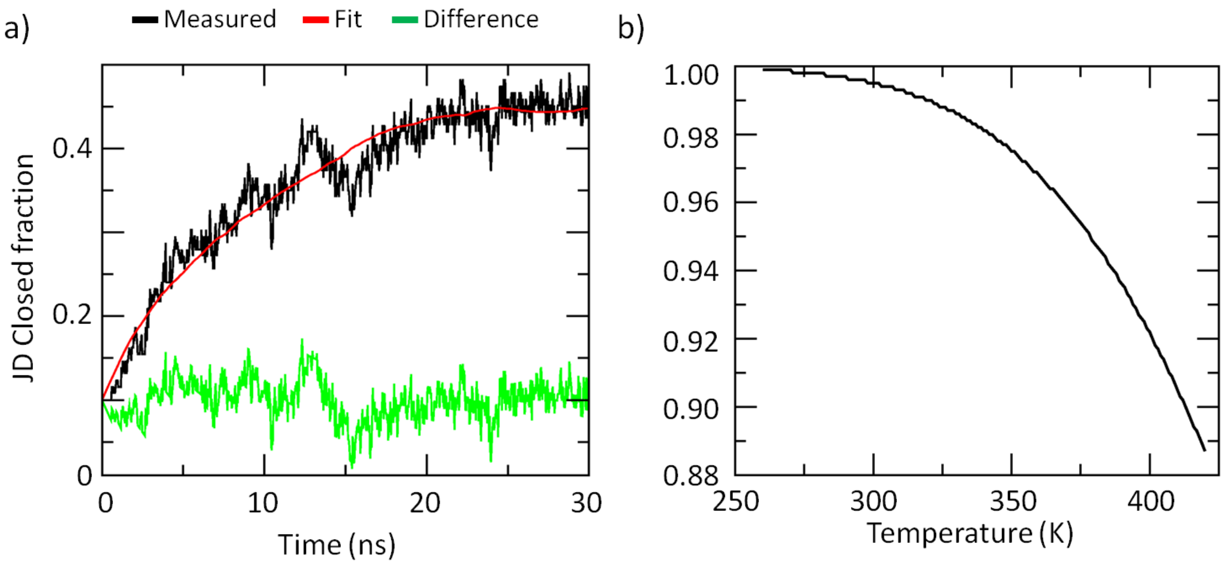

Figure S3. a) Fraction of closed JD as a function of time (black curve), averaged over the first 30 ns of all 128 REMD simulations, compared with the fit (red curve) obtained by minimizing X^2 (Equation 5 of the main text). b) The represented melting curve reports the fraction of closed JD as a function of temperature.

S1.3 Kinetics Estimation of the Josephin Domain Open-to-Closed Transition by Unbiased Molecular Dynamics

The 1YZB[1,2] model was solvated in a dodecahedron box where the minimum distance between the protein and the edge of the box was 1 nm, resulting in a molecular system of about 40000 interacting particles. The net charge of the system was neutralized by the addition of Cl^- and Na^+ ions. The system was first minimized by 1000 steps of the Steepest Descent energy minimization algorithm and then equilibrated at a temperature of 310K [11] and 1 atm[12]. Then, 100 replicas of the system were created by associating to the system atoms initial random velocities from a Maxwell-Boltzmann distribution at 310K. For each system a 20 ns unbiased MD was carried out for a total sampling time of 2 µs.

The analysis of the scatter plot reporting each unbiased MD trajectory snapshot (Figure S4a) show the JD configuration sampled by unbiased MD in term of Radius of Gyration and α3-α5 distance. The kinetic description developed by Van Der Spoel and coworkers[10] was then applied to the MD trajectories for evaluating the folding kinetic constants. In detail, the red curve of Figure S4b was optimized according to Equation 5 reported in the main manuscript.

Throughout the simulated time (20 ns) by unbiased MD simulations, only transitions from open to intermediate (O-I) and from intermediate to closed (I-C) arrangement were sampled. The fraction of JD closed arrangements reported as function of simulated time (Figure S4b).

In agreement with kinetics reported in Figure 3 of the main manuscript and unbiased MD of our previous work[9], C-I and I-O transitions have not been detected throughout the 20 ns of the 100 MD. For this reason, in case of unbiased simulations, we were able to estimate only the forward rate constant (O-I and I-C).

The above mentioned kinetic data, reported in Figure S5 ($\tau_{O\text{-}I}$=88.4 ns and $\tau_{I\text{-}C}$=5.8 ns), are in close agreement with the kinetic rate constants calculated by REMD simulations.

Agreement between biased and unbiased simulations can be considered as a proof of the reliability of the kinetic estimation provided in the present work.

Supporting Information to Thermodynamic and Kinetic Stability of the Josephin Domain Closed Arrangement: Evidences from Replica Exchange Molecular Dynamics

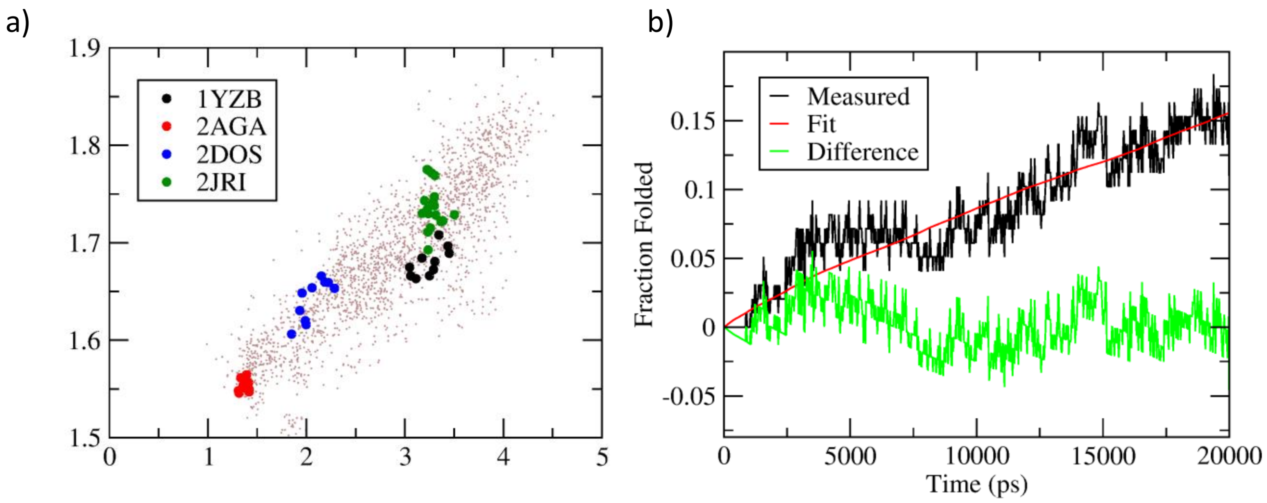

Figure S4. a) Each snapshot, taken from Classical MD trajectory at 310 K, is reported in figure in term of Radius of Gyration and α3-α5 distance. The NMR snapshots derived from the JD models available in literature are colored in red (2AGA), blue (2DOS), black (1YZB) and green (2JRI). b) Fraction of closed JD as a fun*ction of time (black curve), ave*raged over 20 ns of all 100 MD simulations, compared with the fit (red curve) obtained by minimizing X^2 (Equation 5 of the main text).

Equilibrium properties at T = 310K

	Activation energies (kJ/mol)	Rate constant (ns)
O-I	10.6 (0.2)	88.4 (13)
I-C	7.2 (0.1)	5.8 (0.4)

Figure S5. Forward rate constants for folding together with the activation energies at 310 K. Error estimates are given in parentheses. The error estimation was done by varying the cutoff for defining the protein folded state as done in *a* previous work[10]. In detail, we performed our kinetic analysis by changing of 0.02 nm the Radius of Gyration threshold to discern between open/intermediate, and intermediate/closed state.

Supporting Information to Thermodynamic and Kinetic Stability of the Josephin Domain Closed Arrangement: Evidences from Replica Exchange Molecular Dynamics

References

1. Nicastro G, Menon RP, Masino L, Knowles PP, McDonald NQ, Pastore A. The solution structure of the Josephin domain of ataxin-3: structural determinants for molecular recognition. Proc. Natl. Acad. Sci. U. S. A. [Internet]. 2005 [cited 2011 Oct 20];102:10493–8. Available from: http://www.pnas.org/cgi/content/abstract/102/30/10493

2. Nicastro G, Habeck M, Masino L, Svergun DI, Pastore A. Structure validation of the Josephin domain of ataxin-3: conclusive evidence for an open conformation. J. Biomol. NMR [Internet]. 2006 [cited 2014 Mar 10];36:267–77. Available from: http://www.ncbi.nlm.nih.gov/pubmed/17096206

3. Zerze GH, Miller CM, Granata D, Mittal J. Free Energy Surface of an Intrinsically Disordered Protein: Comparison between Temperature Replica Exchange Molecular Dynamics and Bias-Exchange Metadynamics. J. Chem. Theory Comput. 2015;11:2776–82.

4. Nguyen PH, Stock G, Mittag E, Hu CK, Li MS. Free energy landscape and folding mechanism of a β-hairpin in explicit water: A replica exchange molecular dynamics study. Proteins Struct. Funct. Genet. 2005;61:795–808.

5. Bergonzo C, Henriksen NM, Roe DR, Swails JM, Roitberg AE, Cheatham TE. Multidimensional Replica Exchange Molecular Dynamics Yields a Converged Ensemble of an RNA Tetranucleotide. J. Chem. Theory Comput. [Internet]. 2014;10:492–9. Available from: http://pubs.acs.org/doi/abs/10.1021/ct400862k

6. Denschlag R, Lingenheil M, Tavan P. Optimal temperature ladders in replica exchange simulations. Chem. Phys. Lett. 2009;473:193–5.

7. Lingenheil M, Denschlag R, Mathias G, Tavan P. Efficiency of exchange schemes in replica exchange. Chem. Phys. Lett. 2009;478:80–4.

8. Deriu MA, Grasso G, Licandro G, Danani A, Gallo D, Tuszynski JA, et al. Investigation of the Josephin Domain Protein-Protein Interaction by Molecular Dynamics. Salahub D, editor. PLoS One [Internet]. Public Library of Science; 2014 [cited 2014 Oct 21];9:e108677. Available from: http://dx.plos.org/10.1371/journal.pone.0108677

9. Deriu MA, Grasso G, Tuszynski JA, Gallo D, Morbiducci U, Danani A. Josephin Domain Structural Conformations Explored by Metadynamics in Essential Coordinates. Briggs JM, editor. PLOS Comput. Biol. [Internet]. 2016;12:e1004699. Available from: http://dx.plos.org/10.1371/journal.pcbi.1004699

10. van der Spoel D, Seibert MM. Protein Folding Kinetics and Thermodynamics from Atomistic Simulations. Phys. Rev. Lett. 2006;96:238102.

11. Bussi G, Donadio D, Parrinello M. Canonical sampling through velocity rescaling. J. Chem. Phys. [Internet]. AIP; 2007 [cited 2012 Dec 4];126:14101. Available from: http://arxiv.org/abs/0803.4060

12. Berendsen HJC, Postma JPM, Van Gunsteren WF, DiNola A, Haak JR. Molecular dynamics with coupling to an external bath. J. Chem. Phys. [Internet]. AIP; 1984 [cited 2012 Dec 4];81:3684–90. Available from: http://link.aip.org/link/JCPSA6/v81/i8/p3684/s1
